# Insured clients out-of-pocket payments for health care under the national health insurance scheme in Ghana

**DOI:** 10.1186/s12913-021-06401-8

**Published:** 2021-05-08

**Authors:** Patricia Akweongo, Moses Aikins, Kaspar Wyss, Paola Salari, Fabrizio Tediosi

**Affiliations:** 1 School of Public Health, University of Ghana, P. O. Box LG13, Accra, Ghana; 2 Swiss Tropical and Public Health Institute (Swiss TPH), Basel, Switzerland, University of Basel, Basel, Switzerland, Socintrasse 57, 4051 Basel, Switzerland; 3 Institute of Pharmaceutical Medicine (ECPM), University of Basel, Petersplatz 1, 4001 Basel, Switzerland

**Keywords:** Ghana, Out-of-pocket, National health insurance scheme, Insured clients, Inequity, Access

## Abstract

**Background:**

In 2003, Ghana implemented a National Health Insurance Scheme (NHIS) designed to promote universal health coverage and equitable access to health care. The scheme has largely been successful, yet it is confronted with many challenges threatening its sustainability. Out-of-pocket payments (OOP) by insured clients is one of such challenges of the scheme. This study sought to examine the types of services OOP charges are made for by insured clients and how much insured clients pay out-of-pocket.

**Methods:**

This was a descriptive cross-sectional health facility survey. A total of 2066 respondents were interviewed using structured questionnaires at the point of health care exit in the Ashanti, Northern and Central regions of Ghana. Health facilities of different levels were selected from 3 districts in each of the three regions. Data were collected between April and June 2018. Using Epidata and STATA Version 13.1 data analyses were done using multiple logistic regression and simple descriptive statistics and the results presented as proportions and means.

**Results:**

Of all the survey respondents 49.7% reported paying out-of-pocket for out-patient care while 46.9% of the insured clients paid out-of-pocket. Forty-two percent of the insured poorest quintile also paid out-of-pocket. Insured clients paid for consultation (75%) and drugs (63.2%) while 34.9% purchased drugs outside the health facility they visited. The unavailability of drugs (67.9%) and drugs not covered by the NHIS (20.8%) at the health facility led to out-of-pocket payments. On average, patients paid GHS33.00 (USD6.6) out-of-pocket. Compared to the Ashanti region, patients living in the Northern region were 74% less at odds to pay out-of-pocket for health care.

**Conclusion and recommendation:**

Insured clients of Ghana’s NHIS seeking health care in accredited health facilities make out-of-pocket payments for consultation and drugs that are covered by the scheme. The out-of-pocket payments are largely attributed to unavailability of drugs at the facilities while the consultation fees are charged to meet the administrative costs of services. These charges occur in disadvantaged regions and in all health facilities. The high reliance on out-of-pocket payments can impede Ghana’s progress towards achieving Universal Health Coverage and the Sustainable Development Goal 3, seeking to end poverty and reduce inequalities. In order to build trust and confidence in the NHIS there is the need to eliminate out-of-pocket payments for consultation and medicines by insured clients.

## Background

Essential health care services remain unavailable to many people in need of care. Half of the world’s population cannot still obtain essential health services and about 800 million people spend about 10% of their household budget on health care expenses, while over 100 million are further pushed into extreme poverty by paying for care out-of-pocket [1]. The proportion of households and patients paying-out-of-pocket for care is growing with about 808 million people estimated globally to have incurred catastrophic health spending in 2010 [1]. This continues to impose huge financial risks on households, especially vulnerable households, potentially pushing such households into a vicious cycle of poverty.

The declaration of Universal Health Coverage (UHC) as a policy for all countries comes timely as UHC seeks to ensure “all people can use the promotive, preventive, curative, rehabilitative and palliative health services they need, of sufficient quality to be effective, while ensuring that the use of these services does not expose the user to financial hardship” [2]. The demand for social protection schemes as a way of mitigating against the effect of out-of-pocket payments and poverty is growing as social protection schemes prove successful in fighting against poverty.

Social protection schemes either voluntary and/or mandatory have been adopted by middle- and low-income countries with the aim of increasing equity in access to health services and providing financial risk protection, especially for the poor [3]. The introduction of financial protection schemes seeks to pool funds to protect individuals against catastrophic health expenditures. Social health insurance reduces household vulnerability to high out-pocket payments for health services in times of illness through reduction in direct medical costs and loss of income due to ill health [2]^.^ Sood et al. [4] indicated that poor households covered under these schemes tend to have reduced mortality, better access to care and reduce health care expenditures. Out-of-pocket payments by eligible households in this same study was also reduced by 64% for hospital admission fees [4].

Similarly, health care access increases for vulnerable groups covered under health insurance than for those not covered. Globally, evidence shows that social health insurance improves access to health care services [3, 5]. There is also evidence that health insurance decreased out-pocket-payments between 16 and 18% in Vietnam with more substantial decreases for low-income households [6–8]. However, out-of-pocket payments among the insured are still high in other countries and this affects access to health care [7].

Ghana introduced a national health insurance scheme (NHIS) in 2003 as a form of health financing mechanism. The NHIS aims at improving financial access to health care to all people living in Ghana irrespective of social status. Despite over a decade and half of the implementation of the NHIS, enrolment unto the scheme remains low. A published study reports coverage of the NHIS to be 35% [9]. A recent study found that being a member of NHIS significantly decreased the probability of unmet medical needs by 15% and that of incurring catastrophic out-of-pocket (OOP) health payments by 7% relative to no enrolment in the NHIS [10].

Over the years the NHIS has faced several challenges that threatens its sustainability. Amongst these challenges are; the huge benefit package, which covers over 95% of the disease burden of Ghanaians has also been one of the highlights as it is observed to have significant implications for the financing of health care under the scheme. Moreover, there are several studies that have reported poor quality of health care under the scheme [11–13] a factor that is believed to contribute to the low level of enrolment among the populace. Worse yet, there is evidence that the insured are treated unfairly at health facilities, partly due to delays in reimbursement of payment to service providers. Therefore, providers give preference to patients that pay out-of-pocket [14]^.^ Also, it was reported that the currently uninsured perceived the quality of health care to be better than the currently insured in terms of waiting time, availability of medicines and so on [14] .

The delays in payment of services provided to clients by providers is mainly due to inefficient financial flows. There are thus widespread unauthorized charges that insured clients are paying out-of-pocket which should not be the case. Macha et al. [15] reported Ghana to have the highest out-of-pocket payments of health care accounting for about 40% of total health care expenditure, followed by Tanzania at nearly 26% and South Africa at about 18%. This, notwithstanding over 70% of both public and private health facilities are credentialed NHIS facilities in Ghana [16] .

Macha et al. [15], further reported that patients whether insured or not, paid out-of-pocket for medicines at pharmacies due to stock-outs in public health facilities or due to poor understanding of what they are entitled to as clients of the NHIS**.** The question however remains, what type of services are these unauthorized charges being made, who pays these charges and how much do they actually pay out-of-pocket?

## Methods

### Study design

This was a descriptive cross-sectional exit health facility survey. It was conducted among patients accessing out-patient care services at different levels of the health system in three regions of Ghana. The three regions selected were the Northern, Central and Ashanti based on the proportion of people who registered with the NHIS in 2017. The study chose these three regions purposively based on the proportion of NHIS membership in these regions. Ashanti region had the most registered number of clients, with moderate registration in Northern region and low registration in Central region. We also selected these regions because they each had a tertiary hospital that provided teaching, training and served as referral facilities for specialist care.

### Data collection methods

Quantitative data collection approach was used to gather information about services patient paid for and how much they paid out-of-pocket. Patients were interviewed at the point of exit of the health care system using structured questionnaire**.** Data for the study was collected from April 2018 to June 2018.

### Sample size and sampling method

An average of 688 out-patients were interviewed in each region. One district per region was first randomly selected and in each selected district, three health centres, the district hospital and two Community-based Health Planning and Services (CHPS) compounds were then randomly selected based on size of attendance at each of these health facilities. The number of patients interviewed at each level of the health system was proportional to the size of out-patient-department attendance of that facility for the previous year. For each day of the study, interviewees were conveniently sampled and interviewed as they exited the health facility from the dispensary. Since each interview lasted 30–35 min it meant that another interviewee could be only conveniently interviewed after every 30 min. This was done daily until the sample size for each facility was achieved.

The number of OPD (Out-Patient Department) attendances at CHPS facilities was much lower than anticipated and thus more interviews were carried out at the health centre levels than in CHPS compounds. Interviews with patients at the teaching hospital in the Ashanti region could not be conducted as they required their own ethical review board review of the proposal and due to time constraints, the interviews were done at the district and regional hospitals in the Ashanti region.

### Data management and analysis

Data was entered using Epidata and analyses were done using STATA Version 13.1. The study sought to establish the proportion of insured patients paying out-of-pocket for health care and to assess the determinants of paying out-of-pocket and the costs of care. The main outcome variable was the proportion of patients insured and paying out-of-pocket. Socio-economic status was measured using the wealth index where household assets and possessions of patients interviewed were used to construct the wealth index. Households were divided into five quintiles with first quintile representing the poorest household and the 5th quintile representing the least poor household. Data on household consumption expenditure was also gathered to calculate the proportion of household expenditure on health care. A multiple logistic regression was run to establish the association between being insured and paying out-of-pocket for health care. The type of services paid for are presented as proportions and percentages while the costs of health care and household consumption are presented as means and totals.

### Ethical considerations

The protocol was reviewed and approved by the Ghana Health Service Ethics Review Committee (GHS-ERC: 012/03/18) before the commencement of data collection. Informed consent both written and oral was obtained from potential participants before the interviews were conducted. For minors, informed consent from parents or caregivers who accompanied them to the health facility was obtained. Informed consent was sought from all adult participants 18 years and above.

The interviewers explained to all study participants the purpose of the study, study procedure, right to withdraw and measures put in place to ensure confidentiality. Participants were told data will be reported in an aggregated format and anonymity will be ensured in storage and publication of the results of the study.

## Results

### Socio-demographic and economic characteristics of respondents

Of the 2066 respondents who participated in the survey 47% were in the age bracket 18–49 years (Table 1). There were more females 76.7% (1591) who accessed health care than males. Most respondents were Akan 61.4% (1273). This was not surprising, as the Ashanti and Central regions are Akan speaking communities. A little over 50% (1099) were married and 9.4% (195) of the respondents were living together. The average household was size 4.7 (Table 1).

**Table 1:** Socio-demographic and economic background of respondents

	Number	Percentage (%)
**Demographic characteristic**		
*Age group (Years)*		
< 18	651	31.4
18–49	967	47.1
50–69	301	14.5
70+	147	7.1
**Total**	2066	100.0
*Sex:*		
Female	1583	76.6
Male	482	23.4
**Total**	2066	100′0
*Ethnicity:*		
Akan	1273	61.4
Mole-Dangme	680	32.9
Other	82	4.0
Foreign	31	1.5
**Total**	2066	100.0
*Marital Status:*		
Married	1099	53.2
Single-never married	504	24.4
Divorce-separated	81	3.9
Widowed	187	9.1
Living together	195	9.4
**Total**	2066	100.0
**Socio-economic characteristic**		
*Level of education:*	605	29.3
Never been to school	605	29.3
Primary	233	10.8
Junior High School/Middle	624	30.2
Senior High School/O & A Level	398	19.3
Tertiary	216	10.4
**Total**	2066	100.0
*Main occupation:*	153	7.4
Agriculture	282	13.7
Professional/secretarial	153	7.4
Sales/services	432	20.5
Skilled manual craftsmanship	198	9.6
Unskilled manual labour	125	6.1
Unemployed	541	26.2
Others (i.e., aged/retired/students)	339	16.5
**Total**	2061	100.0
*Remittance from relatives:*		
Received remittance	1094	52.7
*Remittance type:*		
Cash Remittance	738	67.6
Gifts/in-kind	353	32.4
**Total**	1091	100.0
*Remittance frequency*		
Weekly/daily	323	30.7
Monthly	249	23.0
Occasionally	501	46.3
**Total**	1073	100.0
*Monthly income (GHS*^*a*^*)*		
< 150	375	31.0
151–300	290	24.0
301–450	127	10.5
451–600	146	12.1
600+	270	22.4
**Total**	1208	100.0

a – Ghanaian local currency, Ghana cedis. GHS1 .00: USD 5.00 (2018)

Less than a third (30.2%) of the participants completed Junior High School with 29.3%(605) without any formal education (Table 2). Those with secondary or higher education were 29.7% (614). Thus, it is not surprising that 26.2% (541) reported being unemployed and with 20.5% (432) in the sales and services industry. Those working in the agriculture sector were 13.7% (282). Those who had retired from active service or aged were 11.7% (241).

Of the 2066 respondents, 52.7% (1094) received remittances from relatives or friends (Table 2). Cash remittances constituted 67.6% (738). Most remittances were received occasionally. The mean monthly income was GHS618.46 (USD123.69) with a median income of GHS300.00 (USD60) for those who reported actively working. Less than a third (31%) of the working population earned between GHS8.00 and GHS150.00 (USD30). Another 24% earned between GHS151–300 (USD30.2–60), showing that 55% of the respondents earned below the minimum monthly wage of GHS 320.00 (USD64.00) for Ghana [17]. The mean monthly household recurrent expenditure of GHS678.00 (USD135.60) was similar to mean monthly income reported by the respondents. Non-recurrent expenditure was slightly higher than the mean monthly recurrent expenditure. 

### Socio-demographic and economic characteristics by region

In all the three regions, most of the respondents were in the 18–49 years age category (Table 2). Those under 5 years of age and less than 17 years were on average 30% (208) in the Ashanti region, 26.6% (181) in Central region and over 37% (255) in the Northern region. This group is exempted from paying NHIS premium thus benefiting from free health care services. Though those 6–17 of school going age are exempted from premium payment, 56.2% of them paid for care OOP. Over 70% of the respondents in each region were females. Women in their reproductive age and who are pregnant are also exempted from paying national health insurance premium. There were variations in the levels of education by region. Close to 50% (339) of respondents in the Northern region had never been to school compared to 23.6% (163) in the Ashanti region and 15% (103) in the Central region (Table 2).

The socio-economic status of respondents in each region showed 45.5% (255) of those in the Northern region to be in the poorest quintile. The Ashanti region reported more patients in the least poor quintile (33.6%) than in Central (18.1%) and Northern regions (5.7%). These differences were statistically significant. However, monthly earnings did not vary much by region. Most participants earned between GHS8–150 (USD1.60–30.00..) and GHS151–300 (USD30.20–60.00.) per month (Table 2).

**Table 2:** Socio-demographic and economic background of respondents by region

Items	Study regions		
	Ashanti (%)	Central (%)	Northern (%)
*Age:*			
< 18	30.0	26.6	37.9
18–49	41.9	47.1	51.2
50–59	10.8	9.2	3.3
60–69	8.8	7.8	3.6
70+	8.5	9.3	4.0
**Total (n)**	692	688	688
*Sex:*			
Female	73.1	77.1	79.9
Male	26.8	22.9	20.1
**Total (n)**	692	688	683
*Level of education:*			
Never been to school	23.6	15.0	49.4
Primary	10.3	13.4	8.7
Junior High School/Middle	34.3	43.3	13.5
Senior High School/O & A Level	22.1	16.9	19.1
Tertiary	9.7	12.3	9.3
**Total (n)**	691	687	687
*Occupation:*			
Agriculture	4.4	17.3	21.0
Professional/secretarial	10.1	8.1	5.4
Sales/Services	23.1	19.1	22.5
Skilled manual craftsmanship	10.7	12.2	7.4
Unskilled manual labour	6.6	10.6	2.1
Unemployed	29.5	22.0	31.1
Other (i.e., retired)	15.7	10.7	10.6
**Total (*n*)**	637	654	672
*Wealth quintiles*			
Poorest	2.2	16.7	45.5
Very poor	9.8	28.7	22.1
Poor	23.2	18.7	17.7
Less poor	31.2	17.8	8.9
Least poor	33.6	18.1	5.7
**Total (*n*)**	682	663	560
*Monthly income (GHS*^*a*^*):*			
< 150	27.3	32.7	32.7
151–300	29.4	21.6	21.7
301–450	10.1	12.0	9.2
451–600	14.2	13.1	8.9
600+	19.1	20.3	27.6
**Total (*n*)**	367	449	392

a – Ghanaian local currency, Ghana cedis

### Utilization of health care services by respondents

Most patients who participated in this survey visited in the last 6 months the government hospital 57.5% (1187) and government health centres 38.2% (790) for health care services (Table 3). The choice of a health facility was based on three main factors; proximity 40.9% (845) of the facility to the patient, NHIS credentialed facility 29.6% (611) and availability of doctors 21.5% (444). Patients reported using hospitals and health centres more for all levels of severity of illness 41.4% (855) with only about 18.6% (384) using these facilities only during severe illness. For severity of illness patients were asked to indicate the stage of the illness (Mild, moderate, severe and very severe) at which they make decision to visit a particular facility to seek care. Patients who presented at the out-patient-department with fever and malaria like signs and symptoms were 47.5% (981) and nearly a quarter of patients 23.4% (485) were there for a review of their condition recommended by the health provider from a previous visit. (Table 3).

**Table 3:** Health Care Services Accessed by Patients

Utilization of health care service	Number	Percentage (%)
**Usual Care source**		
Health Centre	790	38.2
Government-Hospital	1187	57.5
Pharmacy-drug	19	0.9
Private-facility	70	3.4
Total	2066	100
**Reason for choice**		
Proximity	845	40.9
Lab	62	3.0
Drugs available	104	5.0
NHIS Credential	611	29.6
Medical Doctors available	444	21.5
Total	2066	100
**Level of Severity of illness for Choice of Health Facility**		
Severity of illness	384	18.6
Moderate/mild	827	40.0
All levels of severity	855	41.4
Total	2066	100
**Signs and symptoms presenting**		
Fever-malaria like	981	47.5
Difficult-in-breathing	203	9.8
ENT-toothache	115	5.6
Injury	104	5.1
Review	485	23.4
Eye infection	103	5.0
Hypertension-diabetes	55	2.6
Skin Infections	20	1.0
Total	2066	100

### Type of health services provided to patients at the out-patient-department

Over 76.6% (1583) of patients reported having consultation with the health care provider while 14.8% (306) came for a resupply of their medication (Table 4). After consultation, 87.3.% (1382) were prescribed at least one drug. Twenty-nine percent (459) were prescribed two drugs and 25.8% (408) were prescribed three drugs. There was an observed pattern in the number of drugs prescribed and number of these drugs that were supplied by the pharmacy or dispensary of the health facility the patient visited. On average 75.6% (686) of all drugs prescribed were supplied by the pharmacy or dispensary of the facility the patient visited (Table 4).

**Table 4:** Type of Services Accessed by Patients

Service Provision	Number	Percentage (%)
**Services received at Facility**		
Maternal-child	68	3.3
Consultation	1583	76.6
Laboratory	44	2.1
Drugs	306	14.8
ENT-Eye-tooth	32	1.6
Hospital Admission	33	1.6
Total	2066	100
**Number of Drugs Prescribed Among those who consulted**		
No drug prescribed	201	12.7
One drug prescribed	247	15.6
Two drugs prescribed	459	29.0
Three drugs prescribed	408	25.8
Four or More drugs prescribed	268	16.9
Total	1583	100
**Number of drugs prescribed and number received by clients with 2 or more drugs**		
Received All 2 drugs prescribed	354	77.1
Received All 3 drugs prescribed	365	79.5
Received All 4 drugs prescribed	188	70.2
Average of all drugs received	686	75.6

However, of those who were prescribed with two drugs, 77.1% (354) of these drugs were supplied by the health facility they obtained care from. For those who were prescribed three drugs, 79.5% (365) received all the three drugs prescribed from the health facility. Of those prescribed with four drugs only 70.2% (188) were supplied with all four drugs (Table 4) in the health facility they obtained care from. Thus, close to a quarter of all the drugs prescribed were not supplied by the health facility the patients visited.

### Insurance status of patients

**Fig. 1:**
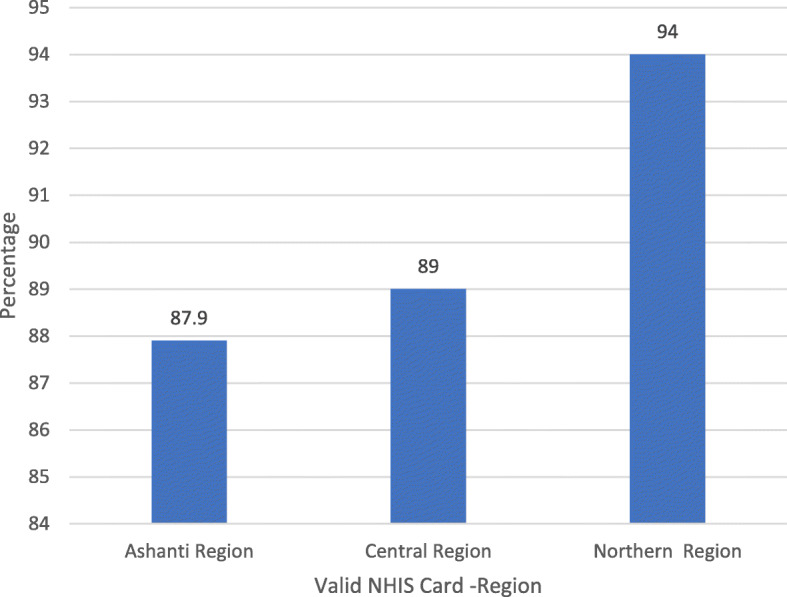
Proportion of Patients with Valid NHIS Card by Region

Many respondents in this survey held a valid NHIS card at the time of the interview. The proportion of all patients with a valid NHIS card was 90.3% (1722/1907) and a similar pattern was observed at the regional levels (Fig. 1). Northern region reported the highest NHIS coverage of 94% and the least (87.9%) coverage was observed in the Ashanti region (Fig. 1). The proportion of males with valid NHIS card was 87.4% (368) while the proportion of females with valid NHIS card was 91.1% (1349) and these differences were statistically significant (*P* < 0.000).

### Out-of-pocket payment for health services

Close to 49.7% (1027) of all patients reported paying for health care at the health facility they visited. Sixty-four percent (657) paid consultation fee and 34.2% (326) paid for drugs (Table 5). Whereas some patients incurred cost that otherwise should have been paid for by the NHIS, other costs were co-payments.

**Table 5:** Out-of-pocket payments for health care by All patients

Payment for Health Care	Number	Percentage (%)
**Paid for services**	1027	49.7
**Services paid for**		
Laboratory investigations	44	4.4
Drugs	326	32.4
Consultation	657	64.0
**Total**	**1027**	**100**
**Cost of care paid for (GHS)**		
1–20	683	66.5
21–40	177	17.2
41–60	77	7.5
61–80	16	1.6
81–100	74	7.2
Total	1027	**100**
**Receipts received for services paid for**		
No receipts given	441	42.5
Receipts for all services	379	36.6
Receipts for some services	217	21.0
**Total**	1027	
Ever paid for services	456	47.7
**Most recent payment**		
Days ago	66	15.9
Weeks ago	322	77.4
Months ago	28	6.7
**Total**	456	100
**Proportion of drugs prescribed patient purchased outside health facility patient visited**	717	34.9
**Reason for purchasing drug**		
Drug unavailable at facility	487	67.9
Not covered by NHIS	149	20.8
Expensive at facility	7	1.0
Don’t know	74	10.3
**Total**	**717**	**100**
**Facility Type and out-of-pocket Payments**		
CHPS	91	40.7
Health Centre	881	42.7
District Hospital	864	54.6
Tertiary	226	51.8

Of those who paid for these services, 42.5% (441) did not get a receipt for the services paid for. Patients reported ever paying for services at the same health facilities. Close to 48 % (456) reported ever paying for these services in previous visits to the health facilities. For the most recent payments, 77.4% (322) paid weeks ago before this survey (Table 5).

Out-of-pocket payment for health care services occurred at all level of health facilities. On average close to 41% (441) of patients who visited the CHPS and 43%(881) health centres paid for health care while an average of 53% of those who visited district, regional and tertiary (teaching training hospitals) health facilities also paid out-of-pocket for health care.

Out-of-pocket payments at district and regional hospitals were slightly higher than those of tertiary hospitals but the differences were not statistically significant (*P* > 0.05).

Of all drugs prescribed, 34.9% (717) were purchased outside the health facility visited (Table 5). The main reasons why the drugs were not supplied by the health facilities patients visited were: unavailability of the drug 67.9% (487) and the drugs prescribed not being covered by the NHIS 20.8% (142).

Most patients (66.5%) paid between GHS1.00 (USD0.20) and GHS20.00 (USD4.00) for consultation and drugs (Table 5).

### Out-of-pocket payment for health services by insured clients

Ninety percent of patients (1722) who accessed health care at all health facilities in the regions had a valid NHIS card and 8% (153) had a valid NHIS card but could not show it at the time of the survey (Table 6).

**Table 6:** Out-of-pocket Payments by the Insured

Health care payment by Insurance Status	Number	Percentage (%)
**Respondents with Valid NHIS card and seen**	1722	90.3
No Valid NHIS Card	178	9.7
Total	1907	**100**
**Paid out of Pocket with valid NHIS card seen**	803	46.9
**Services paid for as NHIS valid card holder**		
Consultation with Valid card seen	1295	75.3
Card not seen	102	68.5
**NHIS valid card purchased drugs**		
Card seen	1079	63.2
Card not seen	111	75.0
**Proportion of NHIS earning income**	998	56.0
**Monthly income by NHIS clients (GHS)**		
8–150	318	31.9
151–300	238	23.9
301–450	101	10.1
451–600	120	12.0
600+	121	12.1
**Total**	**998**	
Average health care costs (GHS)	33.1	
Average Income of NHIS Clients (GHS)	574.7	
Average Household size of NHIS clients	4.7	

Insured clients who paid for health care were 46.9% (803) compared to 49.7% (89) of those who reported not having enrolled with the NHIS. This difference was not statistically significant .

Of those who held a valid NHIS card that was seen, 75.3% (1295) paid for consultation and 63.2% (1079) paid out of pocket for drugs, and 26% paid for both consultation and drugs (Table 6). Of those with valid NHIS cards who could not present them, 68.5% (102) paid for consultation and 75% (111) paid for drugs (Table 6).

The proportion of NHIS clients in this study earning income was 56% (998). The median monthly income of NHIS clients was GHS300.00 (USD60.00) while the mean income was GHS574.72 (USD114.94). However, over 55.6% of NHIS members earned in the range of GHS8.00 to GHS300.00 (Table 6). The average monthly health care costs paid per client was GHS33.1 representing 11% of the median income of GHS300.00 (USD60.00) of patients.

**Fig. 2:**
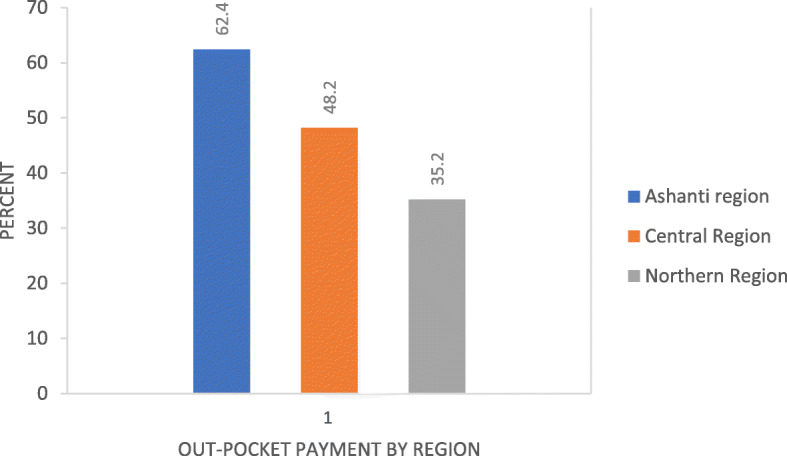
Out-of-Pocket Payment by Region for Insured Clients

NHIS clients paying-out-of-pocket for health care varied by region with 62.4% (431) of those from the Ashanti region paying out of pocket with much lower payments observed in the Central 48.2% (335) and Northern regions 35.2% (238) and these differences were statistically significant (*P* < 0.001) as shown in Fig. 2.

**Fig. 3:**
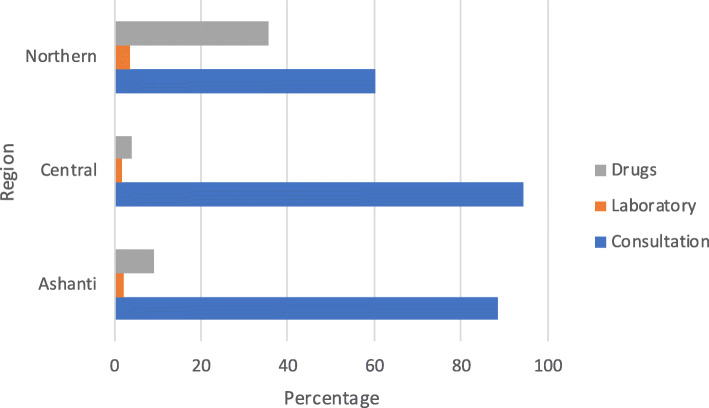
Type of Services Paid Out-of-Pocket by Region (%)

There were regional differences in type of services paid for. Most patients in the Ashanti and Central regions paid more for consultation while patients in the Northern region paid more for drugs (Fig. 3). The differences in type of services paid out-of-pocket by region were statistically significant (*P* < 0.001).

**Fig. 4:**
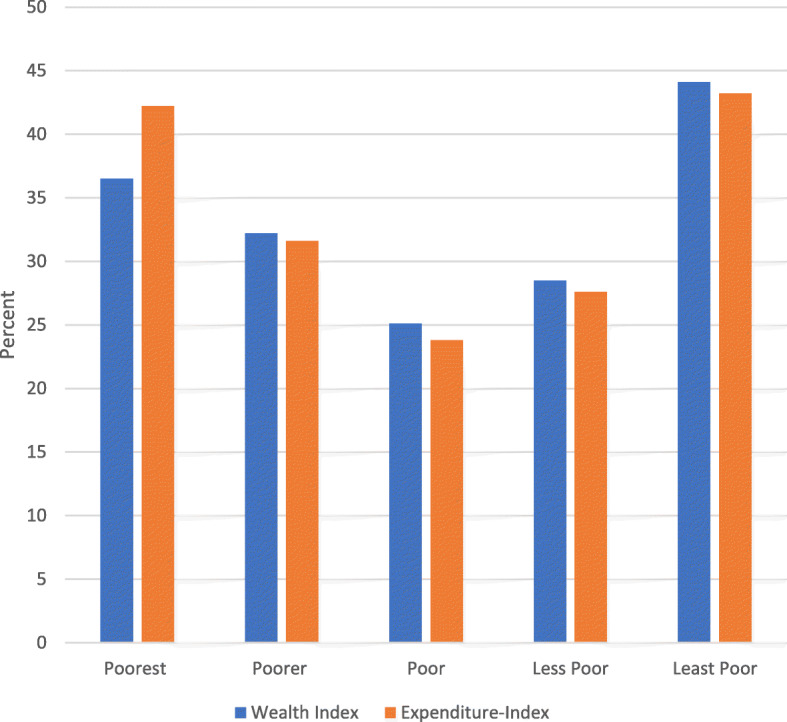
Out-Of-Pocket Payment by Socio-economic Index

The distribution of out-of-pocket payments by socio-economic index showed that the proportion of the poorest that are paying out-of-pocket is similar to that of the least poor. Using both the wealth index generated using household assets and possessions and the monthly household expenditure, both indices showed that 42% of the poorest quintile paid out-of-pocket and a similar proportion 43% of the least poor quintile paid out-of-pocket (Fig. 4) for health care. Thus, though the average paid out-of-pocket was GHS33.00 with 55% paying in the range of GHS1.00–20.00 (USD0.20-USD4.00) and GHS21.00–40.00, (USD4.2–8.00) many of the poor earned between GHS8.00–150.00 (USD1.6–30.00). Thus, the out-of-pocket payments are borne more by the poorest quintile who earned in this range.

### Multiple logistic regression of the determinants of out-of-pocket payments for health care

Sex, age, the level of education were not statistically significant determinants of out-of-pocket payments at the 5% level of significance (Table 7). Region was a statistically significant determinant of out-of-pocket payments. Thus, compared to the Ashanti region, patients living in the Central region had 64% reduced odds (CI: 0.25–0.50) of paying out-of-pocket for health care. Similarly, those living in the Northern region had about 74% reduced odds of paying out-of-pocket compared to patients in the Ashanti region. The odds of paying out-of-pocket increased significantly (33.97; 12.96–89.09) for those receiving laboratory investigation and consultation (12.09; 5.28–27.67). Also, the odds of paying out-of-pocket for health care services were 1.55 times higher for poor households than the poorest households whereas the odds of paying out-of-pocket for health care services were 2.08 times higher for the least poor households than the poorest households (Table 7).

**Table 7:** Predictors of Out-of-Pocket Payments

Variable	Odds ratio	*P*-Value	CI
Sex			
Female (B)			
Male	1.15	0.277	0.89–1.48
Age group of Patient			
Less than 6 years (B)			
Age 6–17	1.8	0.406	0.79–1.78
Age 18–49	1.3	0.131	0.93–1.72
Age 50–59	1.2	0.272	0.78–1.88
Age 60–69	0.83	0.454	0.53–1.33
Age 70 and above	1.04	0.853	0.65–1.66
Level of Education			
Never been to School (B)			
Primary	1.4	0.080*	0.96–2.10
JHS/Middle	1.1	0.429	0.83–1.53
SHS/O/A Level	1.23	0.223	0.88–1.72
Tertiary	1.22	0.343	0.81–1.85
Region			
Ashanti (B)			
Central	0.34	0.000**	0.25–0.45
Northern	0.27	0.000**	0.19–0.38
Illness severity			
Severe (B)			
Moderate	0.75	0.077*	0.53–1.03
Mild	0.66	0.031**	0.45–0.96
All forms severity	0.83	0.216	0.62–111
Type of services received			
OPD-Folder vital statistics taken (B)			
Laboratory investigation	33.97	0.000**	13.0–89.09
Consultation	12.09	0.0000**	5.28–27.66
Drugs	8.06	0.000**	3.33–19.51
Signs-Symptoms presented			
Malaria-like (B)			
Difficult-Breathing	0.99	0.987	0.69–1.43
ENT-toothache	0.59	0.030**	0.36–0.95
Injury	1.78	0.027**	1.06–2.96
Review	0.44	0.000**	0.33–0.60
Eye-infections	0.42	0.001***	0.25–0.70
Hypertension-Diabetes	0.62	0.173	0.31–1.23
Skin Infections	0.45	0.183	0.14–1.46
Poorest (B)			
Poorer	1.17	0.370	0.83–1.64
Poor	1.55	0.012**	1.11–2.18
Less Poor	2.00	0.000**	1.40–285
Least Poor	2.08	0.000**	1.43–3.02

B is the base category; *p < 0.1 (10% level of Significance); **p < 0.05 (5% level of Significance)

## Discussion

The objectives of this study was to determine the services NHIS clients pay for, the actual amounts clients paid out-of-pocket, those paying out-of-pocket and the determinants of out-of-pocket payments. Out-of-pocket payments are occurring at all the levels of health facilities. In lower level health facilities, patients made out-of-pocket payment as much as 40% and in hospitals as much as 53%. Close to 47% of NHIS clients with valid card reported out-of-pocket payments for Out-Patient-Department (OPD) services in this study. Over two-thirds of these patients presented with fever and malaria-like signs and symptoms. This is consistent with the report of Ghana Health Service where malaria was the leading cause of admission in hospitals nationwide in 2018 [18] .

Clients pay for consultation (75%) and drugs (68.5%), the basic services that NHIS covers. The average amount they paid out-of-pocket is GHS33.00. Nonetheless, 55% pay between GHS1.00 and GHS40.00. This level of out-of-pocket payment is confirmed in previous studies where NHIS clients paid between an average of GHS13–17.50 out-of-pocket when seeking treatment [13, 19]^.^ Similarly, a reported 7–18% of insured households incurred catastrophic health expenditure in the Eastern and Central regions of Ghana [20]^.^ Moreover, in this study as many as 48.6% of clients report ever paying for health care in previous visits, further implying that paying out-of-pocket does occur. Patients pay 11% of their median monthly income of GHS300.00 as out-of-pocket payments. As much as 42% of the poorest quintile and 43% of the least poor both pay out-of-pocket for health care, yet the poorest earn less than the current monthly minimum wage in Ghana (GHS320.00).

There were regional differences in out-of-pocket payments with those in the Ashanti region reporting more out-of-pocket payments than those in the Northern region. The variation reported between the two regions could be attributed to the general poverty level variations between these two regions [21]^.^ Generally, the Ashanti region is considered to be more economically vibrant than the Northern region, a situation that puts the people in Ashanti region in a better economic position than their counterparts in the Northern region. Therefore, service providers will be more inclined to expect out-of-pocket payments from the clients in Ashanti compared with clients in the Northern region.

Paying for services is attributed to unavailability of prescribed drugs or prescribed drugs not covered by the NHIS. The hospitals usually procure medical supplies and drugs on credit and pay suppliers later. This is a common practice based on the original design of the NHIS which reimburses service providers some weeks after service provision, when claims have been submitted to the National health insurance authority for payment [22]^.^ However, the chronic delay in claims payment means regular stockout of drugs or deliberate reluctance of service providers to give drugs to NHIS patients who visit their facilities [23–25] Therefore, as a coping strategy, service providers often make NHIS clients pay for drugs in order to keep their health facilities operational. The reasons for paying for consultation fees were to cover administrative expenses of the health facilities.

A range of household characteristics were found to be the key predictors of paying out-of-pocket for health care services. Households in the poor and the least poor category were strong predictors of incurring out-of-pocket health payments. This finding is consistent with previous studies conducted in Sub-Saharan Africa which reported that the burden of out-of-pocket health payments are concentrated among better-off households [26–28]^.^ This is because households who are better off are more predisposed to make such health cost payments unlike the poorest households who are likely to forego health care services when faced with a health risk [26, 29]

Whereas payments by the insured for services or drugs covered by the NHIS is illegal, challenges with the scheme foster these kinds of behaviors. For instance, the delay in reimbursement of health facilities, the unavailability of medicines at health facilities etc. as highlighted above prompt “under the table” payments for health care [23–25]^.^ Also, it is unclear what the boundaries are for health care providers to charge for services. It is also not known whether NHIS clients are aware of services that are not covered by the NHIS. Forty-two percent of the poorest registered with the NHIS reported paying out-of-pocket payments as well. This could be a deterrent to future use of health care or worse yet, contribute to low enrolment or non-renewal of NHIS memberships.

Health insurance presents an alternative to paying out-of-pocket for services and studies showed improved access to health care for all socio-economic groups. It is however becoming clearer that patients are still paying for health services that are supposed to be in the health insurance benefit package. In South Korea, Lee and Shaw [30] reported out-of-pocket payments with health insurance ranging from 65 to 75% for the poorest quintile. This is much higher than in this study where 42% of the insured poorest quintile pay out-of-pocket for health care, nonetheless, it is substantial for the poorest. Kanmiki et al. [31] also reported that between 2010 and 2014 out of pockets payments for health services and medications for primary care declined by 63 and 62% respectively using a 5 year panel data from the Upper East Region of Ghana implying out-of-pocket payments was in the range of 37 and 38% within the same period.

The direct out-of-pocket payments of NHIS beneficiaries continue to be substantially stable and higher relative to household incomes of clients. Direct out-of-pocket payments for the insured in Ghana is not supported by the NHIS [21] and yet it is clear that, the burden of out-of-pocket payments remains on the clients and substantially higher for those in the poorest quintile relative to their earnings.

## Conclusion and recommendation

Insured clients of Ghana’s NHIS seeking health care in credential health facilities make out-of-pocket payments for consultation and drugs that are covered by the scheme.

The out-of-pocket payments are largely attributed to unavailability of drugs at the facilities while the consultation fees are charged to meet the administrative costs of services. These charges occur in disadvantaged regions and in all health facilities.

The high reliance on out-of-pocket health payments can potentially impede Ghana’s progress towards achieving Universal Health Coverage and Sustainable Development Goal 3 (at least 80% essential health services coverage for the entire population of the country and 100% financial protection by the year 2030) seeking to end poverty and reduce inequalities. In order to build trust and confidence in the NHIS there is the need to eliminate out-of-pocket payments for consultation and medicines by clients.

## Data Availability

The data is part of an ongoing study. In view of that we are restricted from sharing the large data set. However, the data contained in this manuscript are available upon a reasonable request to the corresponding author.

## Abbreviations

CHPS: Community-based Planning Services
GHS: Ghana Cedis
NHIS: National Health Insurance Scheme
OOP: Out-Of-Pocket
OPD: Out Patient Department
UHC: Universal Health Coverage
USD: United States of America
